# Keeping the living well

**DOI:** 10.2471/BLT.15.030515

**Published:** 2015-05-01

## Abstract

Few people have done so much to put health research for developing countries on the international agenda. Ade Lucas talks to Fiona Fleck.

“My question was: ‘Why be specialists in the resuscitation of the dead, if we can keep the living well?’”

Q: What made you go into medicine?

A: Originally, I wanted to become a lawyer, to be tried for sedition and to go to a British jail. When I was 10, I had malaria with a high fever, and I didn’t pass urine for three weeks. The whole family was in uproar and they called my uncle, who was the medical officer of health in Lagos. My mother was crying, my father was sombre, but when we heard the horn of his car, a sense of calm came over the house. He treated me with mepacrine and when I saw the power he displayed, I no longer wanted to be a lawyer but to become a doctor.

Q: How did the colonial-era environment shape your approach to life?

A: When I lived in Norwich, in the United Kingdom, for a few months to acclimatize before taking up my studies in Newcastle in October of that year, some of my classmates at the polytechnic in Norwich were so filled with racial prejudice they would cross the road, rather than being seen talking to an African. Then I moved to Newcastle, where the people were generous and hospitable, and where I found wonderful friends. There were lots of black people there and the university was used to hosting West African students. Perhaps these experiences drove me to do well at college, to show them that I was not stupid. If I’d studied medicine in Nigeria, I may not have had the same drive to do so well.

Q: You started out wanting to be a good clinician, why did you switch to public health?

A: My post-graduate training was in internal medicine at Queens University in Belfast in 1957, where my mentor, Graham Bull, said: “Why don’t you audit the courses in epidemiology and statistics? Those are useful tools to have, even for an internist.” I did and then he said: “Why don’t you register for a diploma in public health?” I sat for the exam in June, 1958 and won the prize. Then they appointed John Pemberton as the new head of the preventive medicine department at Queens. He was the co-founder of the International Epidemiological Association (IEA) and when I went home to practise in Nigeria, he invited me to an IEA scientific conference in Korcula, Serbia and Montenegro in 1961. That was a turning point, because I soon became a member of the council and the executive committee of the association and, later, its president. Everything conspired to move me away from my original intention of becoming a good bedside clinician to becoming a public health person.

Q: Why did you become so deeply involved in public health, which you describe in your biography as an unpopular specialization at the time?

A: When I came home to Nigeria in 1960, I was a senior registrar at the teaching hospital in Ibadan. I was struck by the fact that there were about 20 cases of tetanus in the whole of England and Wales a year, whereas at the teaching hospital in Ibadan, there were about a 100 cases a year in the adult medical ward, with 50% of patients dying and yet this disease could be prevented with an inexpensive vaccine. I realized people were dying and ill with diseases that could easily be prevented in Nigeria and that spurred me to pursue a career in public health. My question was: “Why be specialists in the resuscitation of the dead, if we can keep the living well?” I wanted to contribute to health in Nigeria. I felt that my research should be on the health conditions that are common in Nigeria. In 1962, I was invited by the vice-chancellor of the University of Ibadan to switch from clinical medicine to public health. After much consideration, I agreed, and within three years, I was appointed as professor and head of the Department of Preventive and Social Medicine at the age of 33.

Q: You were the first head of the board of the Nigerian Medical Research Council, how did you persuade your peers that research was important?

A: People thought research was a luxury, something you do in a rich country. There was this fable that you must apply the knowledge you have now, rather than seeking new knowledge. I kept saying that research would provide answers to important questions for which the scientific evidence was not available. I became very interested in research and did some in the department of medicine and, when I headed the department of public health, I encouraged my colleagues to do research. We became known. We got into arguments including a famous one with the London School of Hygiene & Tropical Medicine over their paper on obstruction of the ureters in children with schistosomiasis in 1966. The London team recommended surgery but we showed that drug treatment could relieve the obstruction. When our findings became widely accepted, WHO changed its global policy on the control of this disease – from the control of snails, the intermediate host of the parasite – to mass chemotherapy in endemic countries.

Q: At the Special Programme for Research and Training in Tropical Diseases (TDR) you developed a new type of collaboration with the pharmaceutical industry. How did you win over sceptics in the public health community?

A: We had to accept that we were not going to develop new and improved drugs for malaria and other diseases unless we could work amicably with pharmaceutical companies. We made this clear to WHO and we got the blessing of the Joint Coordinating Board of TDR to go ahead, as long as we could show that we were not engaging in any unethical behaviour. We found that if we talked to people within the industry, firms such as Merck, they were prepared to work with us under conditions that were acceptable and ethical. For example, we worked on mefloquine, which was discovered by the US Department of Defence, but the manufacturing costs were so high, it was not used and lay on the shelf. Then chemists from Swiss company Hoffmann La Roche found cheaper ways of synthesizing it. We collaborated on this and did the clinical trials until it was registered. Part of the bargain was that the company provided the drug at low cost for public use in developing countries. That was one example of getting a good price without making any compromises. We then worked on drugs for leprosy with a consortium of drug companies that produced a drug treatment that was ready for testing. We found that this multiple-drug combination worked very well and the companies donated the drugs. At the time there were 122 countries with a significant leprosy problem, today only about three countries have a leprosy problem. So our successes in securing life-saving drugs at low or no cost also helped convince the sceptics of the value of TDR’s work.

Q: Some say that your greatest achievement at TDR was to persuade Merck to donate ivermectin for river blindness (onchocerciasis) free of charge in 1986. How did you do it?

A: That’s an exaggeration. I reviewed the scientific reports showing that the drug was good for river blindness – though not perfect – the question was cost. It had been developed for use in cats and dogs, and cost about US$ 10–15 a dose. In February 1986, I told the then chief executive officer Roy Vagelos that it was too expensive. We had already worked with Merck on a lot of the clinical trials in Africa for the drug and I said that I hoped Merck would give us some concession on the price. I was preparing to argue the case, but before I could, he said that the company would donate the drug. I said, “How much?” He said, “As much as required for as long as it takes.” Finally, in June 1986, Merck sent a telex to WHO and the Joint Coordinating Board during their annual meeting, saying that his company had decided to make it available at no cost to patients and their governments. And they kept to their word. 

Q: Some of TDR’s grant awards sparked controversy, such as grants to researchers in what was then Burma. How did you counter resistance?

A: Some governments disapproved of certain grant recipients, but at TDR we worked through WHO and so we had the right to work with all Member States. Still, we had problems with some individuals. Some countries insisted that the government should nominate grant recipients. We refused, saying that it had to be about science not political favours. Sometimes we used diplomacy to overcome their objections: we gave the government the profile of the person we wanted. Then the ministry of health would call the WHO representative in the country and ask us who WHO had in mind.

Q: How did you became involved in some of the first initiatives to address maternal mortality in the 1980s?

A: At the Carnegie Corporation of New York I was able to support programmes aimed at reducing maternal mortality. We helped to finance the first international conference on Safe Motherhood in Nairobi, Kenya in 1987 as well as health systems research led by a team from the Columbia School of Public Health with a network of 11 centres in West Africa. We also supported training programmes for specialists in obstetrics and gynaecology in Ghana. Little improvement could be seen after the first decade globally, but more recently there has been a dramatic fall in maternal mortality in most developing countries.

Q: Malaria eradication efforts have come full circle since the Global Malaria Eradication Programme was launched in the 1950. In 1973 you chaired the WHO Expert Committee on Malaria that concluded that malaria eradication was not feasible in the short term. What do you think of the recent renewed drive to eradicate malaria by the Gates Foundation?

A: Technology has advanced. I know Bill Gates and have worked with him. I am delighted that he is pushing for this. What we have in malaria is a challenge for which existing tools are marginally effective. If you start with chemotherapy sooner or later you will have to deal with drug resistance. If you start with bednets, people may not use them. If you have a vaccine – this may be more effective than the tools we have now – and if you combine this with other interventions, you can achieve wide coverage and eradication will be possible. Progress is being made and one vaccine is currently being considered for licensing. There are also new ideas about vector control. The most recent one is genetically modifying mosquitoes so that all of their offspring are males, who don’t bite or produce offspring. Eventually we will get there.

